# The Cost of Neurodevelopmental Disability: Scoping Review of Economic Evaluation Methods

**DOI:** 10.2147/CEOR.S370311

**Published:** 2022-10-18

**Authors:** Sanjeewa Kularatna, Amarzaya Jadambaa, Sameera Senanayake, David Brain, Nadia Hawker, Nadine A Kasparian, Bridget Abell, Benjamin Auld, Karen Eagleson, Robert Justo, Steven M McPhail

**Affiliations:** 1 Australian Centre for Health Services Innovation and Centre for Healthcare Transformation, School of Public Health and Social Work, Queensland University of Technology, Brisbane, QLD, Australia; 2 Metro South Health, Queensland Health, Brisbane, QLD, Australia; 3 Cincinnati Children’s Center for Heart Disease and Mental Health, Heart Institute and the Division of Behavioral Medicine and Clinical Psychology, Cincinnati Children’s Hospital Medical Center, Cincinnati, OH, USA; 4 Department of Pediatrics, University of Cincinnati College of Medicine, Cincinnati, OH, USA; 5 Queensland Paediatric Cardiac Service, Queensland Children’s Hospital, Brisbane, QLD, Australia; 6 Digital Health and Informatics Directorate, Metro South Health, Brisbane, QLD, Australia

**Keywords:** neurodevelopmental disorders, attention-deficit/hyperactivity disorder, decision analytic models, economic evaluation

## Abstract

The provision of effective care models for children with neurodevelopmental delay or disability can be challenging in resource constrained healthcare systems. Economic evaluations have an important role in informing resource allocation decisions. This review systematically examined the scope and methods of economic models evaluating interventions for supporting neurodevelopment among children with common neurodevelopmental disorders and identified methods of economic models and presented policy implications. This scoping review employed the Arksey and O’Malley framework and aligned with the Preferred Reporting Items for Systematic Reviews and Meta-Analyses extension for Scoping Reviews (PRISMA-ScR). Four electronic databases were systematically searched to identify eligible model-based economic evaluations of neurodevelopmental care models published since 2000. The Consolidated Health Economic Evaluation Reporting Standards (CHEERS) checklist was used to assess quality of reporting. Data were systematically extracted, tabulated, and qualitatively synthesised across diagnostic categories. Searches identified 1431 unique articles. Twelve studies used a decision analytic model to evaluate care for neurodevelopmental disorders and were included in the review. Included studies focused on attention-deficit/hyperactivity disorder (ADHD, n=6), autism spectrum disorder (ASD, n=3), cerebral palsy (n=2), and dyslexia (n=1). The most used decision analytic modelling approach was a Markov model (n=6), followed by a decision tree (n=3), and a combination of decision tree and Markov model (n=3). Most studies (n=7) adopted a societal perspective for reporting costs. None of the reviewed studies modelled impact on families and caregivers. Four studies reported cost-savings, three identified greater quality of life, and three identified cost increases.

## Introduction

Neurodevelopmental delay or disability (NDD) is a significant concern in the care of children across a range of paediatric specialties and often requires long-term, resource intensive multi-disciplinary intervention.^1^ Relatively common, non-neurological childhood conditions are also independent risk factors for neurodevelopmental delay or disability, including congenital heart disease and prematurity.^2^,^3^ As experts from a range of disciplines work toward addressing the increasing burden associated with neurodevelopmental delay, economic evidence is likely to aid policy and resource allocation decisions to support the implementation of effective screening and intervention for those experiencing, or at risk of, neurodevelopmental delay.

Current healthcare, welfare, child protection, social support, education, and justice systems are complex and characterised by finite budgets, increased demand for services, and high expectations for favourable outcomes.^4^ In this context, decision-makers are required to balance constrained budgets with increasing demand for services.^4^ Economic evaluation provides evidence to inform health system financing based on likely value for money; however, in the complex field of neurodevelopment, the scope of economic evaluation methodologies employed to estimate likely costs and effects of interventions have not been systematically investigated. There is also no “gold-standard” modelling approach for care related to neurodevelopment where potentially costly early intervention may yield substantial benefits across many domains over a lifetime. Economic evaluation methodologies are important to consider in this context, as findings arising from economic modelling are dependent on the modelling structure and approach, including input parameters.^5^

Neurodevelopmental disorders are described as separate entities in the Diagnostic and Statistical Manual of Mental Disorders – 5th Edition (DSM-5) and the International Classification of Diseases 11th Revision (ICD-11). However, developmental delay may be categorised broadly in terms of functional development domains, including fine and gross motor skills, speech and language, attention, visual-spatial integration, memory, learning, social cognition, executive function, and adaptive skills. According to clinical and epidemiological studies, NDDs may entail comorbidities. In practice, children are frequently seen with a combination of delays or impairments across different domains and require a multidisciplinary model of care. Common neurodevelopmental disorders characterised by patterns of delay across multiple functional domains include, but are not limited to, attention-deficit/hyperactivity disorder (ADHD), autism spectrum disorder (ASD), and cerebral palsy. In terms of prevalence, ADHD affects 7.2% and ASD 1% of children worldwide.^6^,^7^ Cerebral palsy prevalence is 2.6% among children in the United States^8^ and China.^9^ While much research has examined screening and interventions for common neurodevelopmental conditions, there has been comparatively less inquiry into the cost-effectiveness of interventions and care models more broadly.^10^

Economic evaluation of interventions for these conditions remains limited relative to the associated negative health and economic burden of neurodevelopmental delay on individuals, families, and societies internationally.^11–14^ The health burden of neurodevelopmental disorders has been estimated using Disability Adjusted Life Years (DALYs)^15^ as well as Quality Adjusted Life Years (QALYs) 10. Overall, 0.38% of worldwide DALYs from all causes, sexes, and ages in 2019 were attributable to ADHD, ASD, and intellectual disability.^15^,^16^ Furthermore, neurodevelopmental disorders often require multiple services, including support for families, accommodation, special educational interventions, social services, and healthcare associated with substantial societal burden. In 2012, the average annual cost of ADHD per child in Europe was estimated to be between €9860 and €14,483.^14^ The total social and economic costs of ADHD in Australia (A$) in 2019 were estimated at A$20.42 billion, including substantial productivity losses of A$10.19 billion.^13^ It has been projected that in the United States (US$) annual direct medical, direct non-medical, and productivity costs combined for ASD will reach US$461 billion by 2025.^17^ Moreover, neurodevelopmental disorders have non-health implications, including low academic achievement,^18^ high rates of unemployment,^19^ and difficulties in social and physical activities^20^ which add to the overall burden of the condition. Additionally, neurodevelopmental disorders can impact primary caregivers’ quality of life, physical and mental health,^21^ employment stability,^22^ and earning capacity, as children with neurodevelopmental disorders often require intensive short- or long-term care, support, and assistance from their family members. Neurodevelopmental disorders often co-exist,^23^,^24^ resulting in a wide range of neurological and psychiatric problems, with assessment, diagnosis, treatment and follow-up care a complex, long-lasting, and costly process. Complex care for children with neurodevelopmental disorders includes multi-disciplinary specialists from a range of disciplines, including psychiatry, psychology, general and developmental paediatrics, speech and language therapy, physiotherapy, and occupational therapy.^1^

Given the importance of this topic, it is encouraging to observe growing interest in economic evaluations of the care of children with neurodevelopmental disorders.^10^,^25–27^ It is now timely to conduct a systematic synthesis of the evidence on economic models in published literature as a potentially rich source of information for informing future economic evaluations of models of care for neurodevelopmental disorders. To date, there has been no attempt to report on the existing decision analytic models with a view to assessing their usefulness for informing healthcare resource allocation decision-making in this context. Thus, the aim of this scoping review was to synthesise current economic evidence regarding care for children with common neurodevelopmental disorders. The quality of included studies was also assessed, with the aim of identifying gaps in the existing literature that remain a priority for further research related to care for children with neurodevelopmental delay or disability.

## Methods

### Study Design

A scoping review was considered the most appropriate study design^28^ to identify the scope of decision analysis methodologies used in economic evaluations of neurodevelopmental disorders, describe the key characteristics of economic models used, and identify key gaps in this field. This design aligned with Arksey and O’Malley’s methodological framework for conducting and reporting scoping reviews^29^,^30^ and included five stages: (1) identifying the research question; (2) identifying relevant studies; (3) selecting studies; (4) collecting data; and (5) collating, summarising, and reporting the results. The review was conducted following a pre-specified protocol (Supplementary Material 1, Tables S1 and S2) and reported according to the Preferred Reporting Items for Systematic Reviews and Meta-Analyses extension for Scoping Reviews (PRISMA-ScR)^31^,^32^ (Supplementary Material 2, Tables S3–S5).

### Stage 1: Identifying the Research Question

A panel (n=8) consisting of clinicians (paediatric-specialist medical, nursing, mental health, and allied health representatives), health services researchers, and health economists was assembled to determine the broad scoping review question through discussions conducted in a mixed mode meeting (combined face-to-face and videoconference). The overarching research question was: *What model parameters and structures have informed decision-analytic models developed for economic evaluations of care for children with common neurodevelopmental disorders?*

The authors subsequently examined published literature to identify the most common childhood neurodevelopmental disorders for review inclusion. Based on the reported prevalence of different neurodevelopmental disorders, the following eight conditions were considered “common neurodevelopmental disorders” (prevalence estimate threshold of more than 0.5% was used): Specific Learning Disorders, Attention-Deficit/Hyperactivity Disorder (ADHD), Communication Disorders, Cerebral Palsy, Motor Disorders, Autism Spectrum Disorder (ASD), Tic Disorders, and Intellectual Disability (Supplementary Material 1).

### Stage 2: Identifying Relevant Studies

To identify studies that had conducted modelled economic evaluations of care for children with neurodevelopmental disorders, four electronic databases were searched: PubMed, PsycINFO (via EBSCOhost), the International Network of Agencies for Health Technology Assessment, and Paediatric Economic Database Evaluation. Searches were conducted up to June 29, 2021 (date of last search) and restricted to English-language documents published since January 1, 2000. This period was chosen to ensure studies were relevant to current and more recent models of care. We received consultation from an experienced medical librarian on identifying and combining different key-terms and subject headings for the search strategy. This was built around the key concepts of “neurodevelopmental disorders” and “model-based economic evaluations”, with appropriate adjacency and truncation settings (see Supplementary Material 1 for full search strategy). Reference lists of included studies were manually searched for other potentially relevant studies and citation chaining. After removal of duplicates using EndNote software, records were imported into a web-based review-management platform, Rayyan.^33^

### Stage 3: Study Selection

The study selection criteria were tested on a sample of abstracts to ensure appropriateness for capturing relevant articles. The study selection process consisted of two levels of screening: title and abstract review, followed by a full-text review. Initially, two investigators (AJ, NH) independently screened the title and abstract of all retrieved citations. In the second step, the same two investigators independently assessed full-texts to determine if each met the pre-specified inclusion/exclusion criteria; those that met the inclusion criteria were included in the review. Uncertainties about eligibility at either screening stage were resolved through discussion, including with a third reviewer (SS).

### Inclusion Criteria

1. Pharmaceutical or non-pharmaceutical treatment, intervention, or follow-up surveillance of children with any of eight common neurodevelopmental disorders (described above);
2. An economic evaluation based on a decision-analytic model, reporting both costs and benefits of the intervention and a comparator;
3. A time horizon of more than 12 months, to capture long-term cost and health outcomes for the models cohort;
4. Written in English; and
5. Published after January 01, 2000.

### Exclusion Criteria

1. Non-model-based economic evaluations;
2. Model-based economic evaluation of screening programmes for the eight defined neurodevelopmental disorders;
3. Modelled a time horizon of less than 12 months; and
4. A protocol, narrative review, letter, commentary, news article, or conference abstract.

Final decisions regarding the inclusion or exclusion of studies were made by consensus between three reviewers (AJ, NH, SS). Full inclusion and exclusion criteria for the selection of studies are shown in Supplementary Material 1.

### Stage 4: Data Collection

In preparation for data extraction, a pre-designed data extraction spreadsheet was piloted and iteratively revised by the research team. Two authors (AJ, SS) extracted data using the final version of this spreadsheet in Microsoft Excel. This included: (a) study characteristics (author, publication year, study population); (b) information on model structures (perspective, intervention, comparator, discount rate, model type, time horizon, input parameters, effectiveness measure, sensitivity analysis, and willingness to pay threshold value); (c) information on health utility values used in the Markov model (health states, utility values and sources); and (d) information on cost-effectiveness analysis (incremental cost, incremental effect, incremental cost-effectiveness ratio [ICER], and policy recommendations).

### Stage 5: Data Summary and Synthesis of Results

To summarise the data, findings were aggregated to provide an overview of the general characteristics of included studies, with detailed information about each model’s structure, including health states, utility values, and information on cost-effectiveness results. Key information related to study characteristics and modelling approaches were also tabulated. Findings were also synthesized and described within diagnostic categories.

### Quality Appraisal

The study reporting quality was assessed using the Consolidated Health Economic Evaluation Reporting Standards (CHEERS) statement.^34^ This 24-item checklist consolidates and updates previous reporting guidelines and consists of recommendations on reporting methods and findings for economic evaluations. The reporting quality assessment for each study is presented in Supplementary Material 4.

## Results

**Figure 1:**
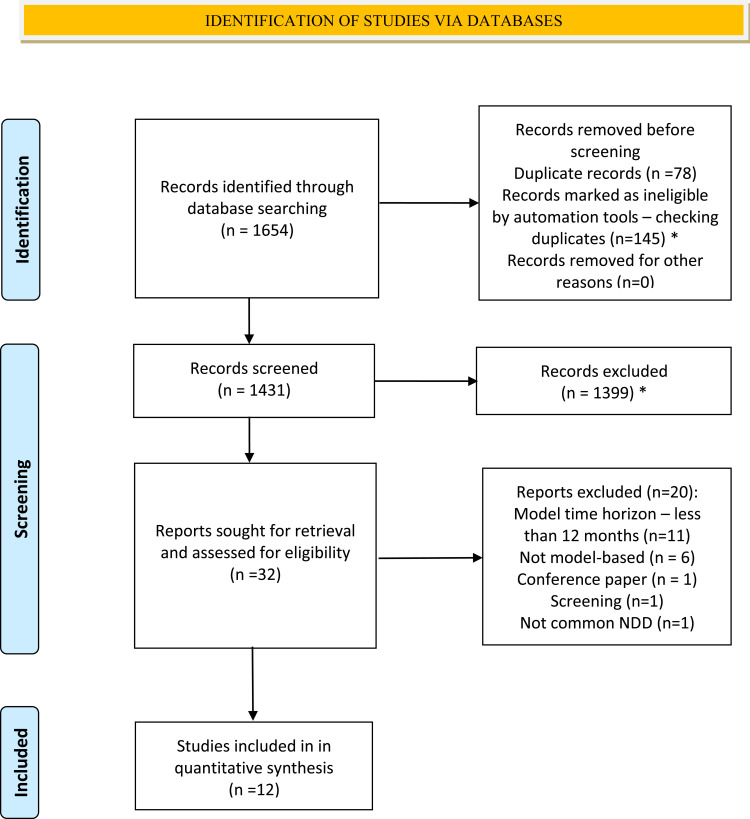
PRISMA flow diagram showing the process of study selection for review inclusion. Adapted from Page MJ, McKenzie JE, Bossuyt PM, et al. The PRISMA 2020 statement: an updated guideline for reporting systematic reviews. BMJ. 2021;372:n71. Creative Commons.

The PRISMA flow diagram for study selection is illustrated in Figure 1. In summary, a total of 1654 citations were identified in the electronic database search, of which 223 were excluded as duplicates. During title and abstract screening, 1399 records were excluded. Of the remaining 32 full-texts, 12 met review inclusion criteria. No further articles were identified by searching the references and citations of the included studies.

### Background Information of Included Studies

Characteristics of the 12 included studies are summarised in Table 1. Included studies conducted evaluations of either clinical management (n=5), pharmaceutical agents (n=4), or behavioural management (n=3) for children diagnosed with ADHD (n=6), ASD (n=3), cerebral palsy (n=2), or dyslexia (n=1). The most used decision-analytic model was a Markov model (n=6), followed by a decision tree (n=3), and a combination of decision tree and Markov model (n=3). None of the included studies used microsimulation or discrete event simulation modelling as the method for analysis. Most evaluations were from the societal perspective (n=7), with time horizons ranging from six years to lifetime. The most used discount rate was 3% (n=6).

**Table 1:** General Characteristics of the Included Studies (n=12)

Item	Specification	Number of Studies
**Study country**	The Netherlands	4
	USA	3
	Canada	2
	Brazil	1
	Spain	1
	UK	1
**Type of economic model**	Decision-tree	3
	Markov model	6
	Decision tree AND Markov model	3
**Study perspective**	Societal only	7
	Health care system only	2
	Provincial/Government/Public and Societal	2
	Health Care System, Public Sector and Societal	1
**Model time horizon**	5–10 years	6*
	11–20 years	5*
	More than 21 years	2
	Lifetime	1
**Neurodevelopmental Disorder** (NDD)	ADHD	6
	ASD	3
	Cerebral palsy	2
	Dyslexia	1
**Intervention classification**	Clinical management or follow-up	5
	Pharmaceutical agent	4
	Behavioural program/management	3
**Health outcome**	QALY	9
	DFLY	2
	LY of serious delinquent behavior prevented	1
**Discounting**	3%	6
	3.5%	1
	4%	1
	Other**	4
**Publication year (CHEERS checklist available 2013)**	Before 2013	3
	After 2013	9

**Notes**: *One study ran model over 6, 12, and 18 years so total exceeds 12;** different discount rates for costs and effects or discounted costs only.

**Abbreviations**: ADHD, Attention Deficit Hyperactivity Disorder; ASD, Autistic Spectrum Disorder; QALY, Quality Adjusted Life Year; DFLY, Dependency Free Life Years; LY, Life Years.

### Model Structures in the Included Studies

To assist in synthesis of findings, the studies were divided into four groups according to the conditions represented: attention-deficit/hyperactivity disorder (ADHD), autism spectrum disorder (ASD), cerebral palsy, and dyslexia (Table 2).

**Table 2:** Information on Model Structures in the Included Studies (n=12)

#	Authors, Year	Study Population/ Sample/Age Range	Perspective	Intervention	Comparator	Discount Rate	Model Type	Time Horizon	Source for Model Input Parameters	Model Input Parameters	Effectiveness Measure	Sensitivity Analyses	WTP Threshold
**1. Attention Deficit Hyperactivity Disorder (ADHD)**													
1	Faber et al, 2008^38^	Youths with ADHD	Societal	Long-acting Methylphenidate Osmotic Release Oral System	Immediate Release Methylphenidate	Costs and effect: 4%	Decision tree followed by Markov model	10 years	Literatures and expert panel opinion	Costs related to (a) treatment options: IR MPH and OROS MPH, and (b) special education; Transition probabilities; Health state utilities	QALY gained	Univariate SA, Scenario analyses	€20,000–30,000/QALY
2	Denchev et al, 2010^39^	7-year-old children with ADHD	Societal	**Strategy 2**: (History and Physical examination normal + ECG) abnormal + Cardiology referral; **Strategy 3**: History and Physical examination + ECG abnormal + Cardiology referral	**Strategy 1**: History and Physical examination abnormal + Cardiology referral	Costs and effects at 3%	Markov model	10 years	Literatures and expert opinion	Costs related to strategies direct and indirect medical costs; the value of patient/parent time associated with treatment; Event probabilities; Health state utilities	QALY gained	OW and TW	$50,000/QALY
3	Schawo et al, 2015^37^	6-year-old children with ADHD	Societal	Methylphenidate Osmotic Release Oral System	Immediate Release Methylphenidate	Costs at 4%, effects at 1.5%	Markov model	12 years	Literature and expert panel data	Costs related to (a) treatment options: IR MPH and OROS MPH, (b) non-medical interventions, (c) special education, (d) criminal justice, (e) low-income support, and (f) caregivers; Transition probabilities; Health state utilities	QALY gained	Scenario analyses	Up to €80,000/QALY
4	van der Schans et al, 2015^40^	Youth with ADHD who were sub-optimal responders to Immediate Release Methylphenidate	Societal	Long-acting Extended-Release Methylphenidate	Short acting IR MPH or Me Immediate Release Methylphenidate Medikinit CR/ Equasym XL	Costs at 4%, effects at 1.5%	Decision tree followed by Markov model	10 years	Literature and expert opinion	Direct and indirect (education) costs, productivity costs of caregivers; Transition probabilities; Health state utilities	QALY gained	Univariate SA and PSA	Not explicitly mentioned
5	Maia et al, 2016 (35)	Children and adolescents with ADHD	Public Health	Immediate Release Methylphenidate	No treatment	Costs at 5%	Decision tree followed by Markov model	6 years	Literature and Delphi panel	Direct costs, transition probabilities, health states utilities	QALY gained	OW and TW	I$11,530/QALY
6	Freriks et al, 2019^36^	Children with ADHD	Societal	1. Medication management, 2. Behavioral treatment, and 3. The combination thereof	A community-delivered treatment	Costs and effects at 3%	A Continuous time Markov model	10 years	Literature	Direct and indirect medical costs and additional costs for attending special education, criminal costs; Transition probabilities, Health states	LY of serious delinquent behavior prevented	Deterministic SA	US$12,370/LY
**2. Autistic spectrum disorder (ASD)**													
7	Penner et al, 2015^41^	Toddlers with undifferentiated developmental concerns	Provincial and societal(Mark et al, 2020)	1. Intensive Early Start Denver Model-I 2. Pre-diagnosis parent delivered Early Start Denver Model - PD	Early intensive behavioral intervention	Costs and effects at 3%	Decision tree	Up to 65 years old	Literature	Intervention costs and costs related to caregivers; Dependent and independent outcomes	DFLYs	OW and PSA	Not explicitly stated
8	Piccininni et al, 2017^42^	Children with severe ASD	Government and societal	1. Intensive behavioral intervention with reduced wait time 2. Intensive behavioral intervention with eliminated wait time	Intensive behavioral intervention with current wait time	Costs and effects at 3%	Decision tree	Up to 65 years old	Literature and estimation	Public funding for education, health, and societal services; costs to caregivers; independent outcomes	DFLYs	OW and PSA	Up to Can$100,000
9	Mark et al, 2020^43^	Preschool children with ASD	Public Health sector, Societal	Early intensive Applied Behavior Analysis based interventions (Early intensive behavioral intervention, EIBI and NDBI)	Treatment as usual or eclectic interventions	Costs and effects at 3.5%	Not explicitly stated (Markov decision model identified)	15.5 years	Literature	Intervention costs, costs of treatment as usual, costs of autism in school, education costs, social care and health care costs, care costs in adulthood, health states utilities	QALY gained	TW, Scenario analysis	£30,000/QALY
**3. Cerebral palsy (CP)**													
10	Vallejo-Torres et al, 2019^44^	Children with cerebral pals	Spanish National Health System	A surveillance program to prevent hip dislocation	Without surveillance program to prevent hip dislocation	Costs and effects at 3%	Decision tree	20 years	Literature and existing regional tariffs	Health system costs: direct and indirect medical costs, Transition probabilities, Health state utilities	QALY gained	OW and PSA	€25,000/QALY
11	Kazarian et al, 2021^45^	Children with Cerebral Palsy aged 7–12	Societal	Surgery	Botulinum toxin injections	Costs and effects at 3%	A Markov transition-state model	Lifetime	Literatures	Direct (surgery, botulinum toxin injection, rehabilitation) and Indirect (parent and caregivers) costs; Health state utilities	QALY gained	OW	$50,000/QALY
**4. Dyslexia**													
12	Hakkaart-van Roijen et al, 2011^46^	Young children with severe dyslexia	Societal	Computer-aided program for diagnosis and treatment (Thedyslexia protocol)	Care as usual for severe dyslexia	Costs at 4% and effects at 1.5%	A markov model	6, 12, and 18 years	Literature, patient files, expert opinion	Direct and Indirect (remedial teaching at primary school) costs, Health state utility	QALY gained	Scenario analysis	Not explicitly mentioned

**Abbreviations**: ABA, Applied Behavior Analysis; IBI With RWT, Intensive Behavioral Intervention with Reduced Wait Time; IBI With EWT, Intensive Behavioral Intervention with Eliminated Wait Time; IBI With CWT, Intensive Behavioral Intervention with Current Wait Time; ESDM-I, Intensive Early Start Denver Model; ESDM-PD, Parent Delivered Early Start Denver Model; EIBI, Early Intensive Behavioral Intervention; NDBI, Naturalistic Developmental Behavioral Intervention; ECG, Electrocardiogram; DFLY, Dependency Free Life Years; H&P, History and Physical Examination; CUA, Cost-Utility Analysis; CEA, Cost-Effectiveness Analysis; CBA, Cost-Benefit Analysis; CCA, Cost Consequences Analysis; MPH OROS, Methylphenidate Osmotic Release Oral System; IR MPH, Immediate Release Methylphenidate; ER MPH, Extended-Release Methylphenidate; QALY, Quality -Adjusted Life-Years; LY, Life Years; OW, One-Way; TW, Two-Way; TAU, Treatment as Usual; MW, Multi-Way; SA, Scenario Analysis; PSA, Probabilistic Sensitivity Analysis; TA, Threshold Analysis; NA, Not Applicable; WTP, Willingness To Pay.

### Attention-Deficit/Hyperactivity Disorder (ADHD)

Six studies reported economic evaluation of care for children with ADHD (Table 2). Of the six, four studies used a Markov model, while the remaining two used a combination of decision tree and Markov models. Most (n=5) were conducted from a societal perspective as payer, with one conducted from the perspective of a publicly funded health system as payer.^35^ The time horizon ranged from 6 to 12 years, with no models run over a lifetime horizon.

The included economic evaluations examined the effectiveness of pharmaceutical interventions (n=4) or clinical management (n=2) for ADHD. Effectiveness was measured using QALYs in all but one study 36. Freriks, Mierau, van der Schans, Groenman, Hoekstra, Postma, Buskens and Cao^36^ used prevented life-years of serious delinquent behaviour as the effectiveness measure for three major forms of ADHD treatment (medication management, behavioural treatment, and the combination of the two) compared with community-delivered treatment. Annual discount rates between 1.5% and 4% were applied to both costs and effects in five of the studies with one study Maia, Stella, Wagner, Pianca, Krieger, Cruz, Polanczyk, Rohde and Polanczyk^35^ discounting only costs at 5%.

Of the six studies, one^36^ performed only deterministic sensitivity analysis, another^37^ performed scenario analysis, while the remaining four studies performed two sensitivity analyses. Faber et al, Annemans and Postma^38^ performed both univariate sensitivity analysis and scenario analyses. One-way and two-way sensitivity analyses were performed in two studies.^35^,^39^ van der Schans et al^40^ conducted both one-way sensitivity and probabilistic sensitivity analysis to examine the robustness of model results to the input parameters used.

### Autism Spectrum Disorder (ASD)

All three studies reporting model-based economic evaluations of ASD examined the effectiveness of behavioural interventions (Table 2). Two of the three studies used a decision tree, with up to 65 years-time-horizon to capture long-term costs and dependency-free life years for the model cohorts from Canadian provincial and societal perspectives.^41^,^42^ Costs and effects were discounted at 3%; one-way and probabilistic sensitivity analyses were performed in both studies. The remaining study used a Markov model to capture costs and QALYs gained over 15 years from UK public health and societal perspectives.^43^ Costs and effects were discounted at 3.5%, with £30,000/QALY used as a threshold of willingness to pay for health benefits.

### Cerebral Palsy

The two studies focussing on cerebral palsy evaluated the effectiveness of clinical surveillance and clinical management for this condition (Table 2). One study used a decision tree over a 20-year time-horizon to capture QALYs gained and costs in the Spanish health system.^44^ The other study used a Markov model with a lifetime horizon to capture QALYs gained and direct and indirect medical costs from the US societal perspective.^45^ Costs and effects were discounted at 3% in both studies. Kazarian, Van Heest, Goldfarb and Wall^45^ performed a one-way sensitivity analysis, whereas the other study^44^ performed one-way and probabilistic sensitivity analyses to assess the potential implications of parameter uncertainty.

### Dyslexia

Only one study evaluated an intervention for children with dyslexia (Table 2). It examined the effectiveness of an educational and behavioural intervention using a Markov model to capture both direct and indirect medical costs and QALYs gained over 6, 12, and 18 years from the Netherlands’ societal perspective.^46^ Scenario analyses were conducted to account for uncertainty in the model’s parameters. A willingness-to-pay threshold was not reported.

### Other Common Neurodevelopmental Disorders

No model-based economic evaluations of interventions designed for children with Communication Disorders, Motor Disorders, Tic Disorders, or Intellectual Disability were found.

### Costing Perspectives

Of the 12 included studies, 10 reported taking a societal perspective and included both direct and indirect medical costs, intervention costs, additional costs for attending special education, and/or some form of caregiver productivity losses, mostly due to absenteeism from work. Three of these studies^41–43^ also reported taking a payer perspective. The remaining two studies reported a public health system^35^ and a Spanish national health service perspective^44^ and included only health system costs.

### Health Utility Values Used in Markov Models

Markov models were used in nine studies either alone (n=6) or in combination with a decision tree (n=3) (Table 3). In three of these studies, which evaluated interventions for children with ASD,^43^ cerebral palsy^45^ and dyslexia,^46^ health states were not explicitly stated. The remaining six studies used a Markov model to evaluate interventions for ADHD. One of these studies developed a continuous-time Markov model based on delinquency states, with prevented Dependency Free Life Years (DFLYs) used as the effectiveness measure.^36^ For the remaining five studies, QALYs were the main effectiveness measure. The utility values to calculate QALYs for the Markov health states were derived from published literature reporting health state utilities for youth with ADHD using the EuroQol-5 dimensions (EQ-5D).^37–40^ For one study, utility was estimated with the Health Utilities Index (HUI) questionnaire and a specific formula provided by HUI Inc.^35^ Reported utility values for children with ADHD varied between 0.65 (treatment stopped) 40 and 1 (functional remission).^38^,^40^

**Table 3:** Description of Markov Model Used in the Included Studies (n=9)

No	Authors, Year	Health States	Utility Values	Sources
**Attention Deficit Hyperactivity Disorder (ADHD)**				
1	Faber et al, 2008^38^	**Eight health states**: Utility weights were assessed with the EuroQol EQ-5D (proxy report based)		
		Immediate Release Methylphenidate (IR MPH):		
		1. Suboptimal response (with insufficient daily exposure to IR MPH)	Mean (SD)=0.901(0.14)	(Secnik et al, 2005)
		2. Optimal response (without adverse effect)	Mean (SD)=0.913(0.13)	(Secnik et al, 2005)
		3. Treatment stopped	Mean (SD)=0.899(0.15)	(Secnik et al, 2005)
		4. Functional remission	1	(Secnik et al, 2005)
		Methylphenidate Osmotic Release Oral System (MPH OROS):		
		5. Optimal response (without adverse effect)	Mean (SD)=0.930(0.11)	(Secnik et al, 2005)
		6. Noncompliance	Mean (SD)=0.899(0.13)	(Secnik et al, 2005)
		7. Treatment stopped	Mean (SD)=0.899((0.15)	(Secnik et al, 2005)
		8. Functional remission	1	(Secnik et al, 2005)
2	Denchev et al, 2010^39^	**Five health states**: Utility weights were assessed with the EuroQol EQ-5D (proxy report based)		
		1. ADHD no treatment	Mean (SD;95% CI) =0.88(0.133; 0.826–0.934)	(Cottrell et al, 2008)
		2. ADHD treated with and responsive to medication, no side effect	Mean (SD;95% CI) =0.93(0.107; 0.907–0.953)	(Cottrell et al, 2008)
		3. ADHD, treated with and responsive to medication, side effect	Mean (SD;95% CI) =0.912(0.124; 0.885–0.939)	(Cottrell et al, 2008)
		4. Remitted ADHD	Mean (95% CI) =0.95(0.93–0.97)	Estimated by Denchev, Kaltman, Schoenbaum, and Vitiello (2010)
		5. ADHD age ≥17 years	Mean (95% CI) =0.91(0.89–0.93)	Estimated by Denchev et al (2010)
3	Schawo et al, 2015^37^	**Nine health states**: Utility weights were assessed with the EQ-5D (both self-report and proxy report based)		
		1. Optimal Patients 8–12 years	Mean (SE)=0.82(0.0979)	(van der Kolk et al, 2014)
		2. Suboptimal Patients 8–12 years	Mean (SE)=0.74(0.01577)	(van der Kolk et al, 2014)
		3. Treatment stopped Patients 8–12 years	Mean (SE)=0.74(0.01588	(van der Kolk et al, 2014)
		4. Optimal Patients 13–18 years	Mean (SE)=0.86(0.00897)	(van der Kolk et al, 2014)
		5. Suboptimal Patients 13–18 years	Mean (SE)=0.77(0.02645)	(van der Kolk et al, 2014)
		6. Treatment stopped Patients 13–18 years	Mean (SE)=0.83(0.01499)	(van der Kolk et al, 2014)
		7. Optimal Caregivers	Mean (SE)=0.85(0.00897)	(van der Kolk et al, 2014)
		8. Suboptimal Caregivers	Mean (SE)=0.83(0.01499)	(van der Kolk et al, 2014)
		9. Treatment stopped Caregivers	Mean (SE)=0.83(0.01499)	(van der Kolk et al, 2014)
4	van der Schans et al, 2015^40^	**Eight health states**: Utility weights were assessed with the EQ-5D (self-report and proxy report based)		
		Immediate Release Methylphenidate (IR MPH):		
		1. Suboptimal treated	Mean (SD)=0.7(0.20)	(Lloyd et al, 2011)
		2. Optimal treated	Mean (SD)=0.82(0.19)	(Lloyd et al, 2011)
		3. Treatment stopped	Mean (SD)=0.65((0.21)	(Lloyd et al, 2011)
		4. Functional remission	1	(Lloyd et al, 2011)
		Extended-Release Methylphenidate (ER MPH):		
		5. Suboptimal treated	Mean (SD)=0.7(0.20)	(Lloyd et al, 2011)
		6. Optimal treated	Mean (SD)=0.82(0.19)	(Lloyd et al, 2011)
		7. Treatment stopped	Mean (SD)=0.65(0.21)	(Lloyd et al, 2011)
		8. Functional remission	1	(Lloyd et al, 2011)
5	Maia et al, 2016^35^	**Three health states**: Utility weights were assessed with the HU questionnaire (proxy report based)		
		Children (6–12 years) and Adolescents:		
		1. Methylphenidate initiated	Children: 0.79; Adolescents: 0.75	Estimated by Maia et al (2016)
		2. Spontaneous improvement	Children: 0.73; Adolescents: 0.69	Estimated by Maia et al (2016)
		3. No spontaneous improvement	Children: 0.69; Adolescents: 0.66	Estimated by Maia et al (2016)
6	Freriks et al, 2019^36^	**Three health states:**		
		No delinquency	Not applicable as the outcome is life-years of serious delinquent behavior prevented	
		Minor to moderate delinquency		
		Serious delinquency		
**Autistic spectrum disorder (ASD)**				
7	Mark et al, 2020^43^	Not applicable as health states were not explicitly stated		
**Cerebral palsy (CP)**				
8	Kazarian et al, 2021^45^	Not applicable as health states were not explicitly stated		
**Dyslexia**				
9	Hakkaart-van Roijen et al, 2011^46^	Not applicable as health states were not explicitly stated		

### Information on Cost-Effectiveness Findings and Policy Suggestions

Evidence on the cost-effectiveness outcomes of the interventions for children with common NDDs are summarised in Table S5, Supplementary Material 3. Most studies were cost-utility analyses (n=9), with the remaining three reporting on cost-effectiveness analyses with other measures of effect. All studies of decision-analytic models reported outcomes using ICERs. Four studies reported the intervention as cost-saving and as improving quality of life for children,^37^,^40^,^42^,^45^ whereas the remaining studies were cost-increasing and improved quality of life for children. Only one study found the ICER results were estimated to be above the National Institute for Health and Care Excellence (NICE) threshold for cost-effectiveness; £30,000 per QALY.^43^ The estimated ICER was £179,799 per additional QALY in the pessimistic scenario in that study and £43,289 per additional QALY in their optimistic scenario,^43^ implying their findings did not indicate the intervention evaluated was likely to be cost-effective.

### Cost-Effectiveness of Pharmaceutical Interventions for ADHD

Although there was substantial intervention heterogeneity across the included studies, three focussed on pharmaceutical interventions for ADHD. These three studies used decision-analytic models to evaluate the cost-effectiveness of a switch from immediate-release methylphenidate (IR MPH) to extended-release methylphenidate (ER MPH) or long-acting methylphenidate osmotic release oral system (MPH OROS) for children with ADHD from the Netherlands societal perspective.^37^,^38^,^40^ The findings showed that ER MPH or long-acting MPH OROS may be considered as cost-effective treatment^38^ as well as cost-saving and more effective treatment^37^,^40^ compared with the immediate-release (IR) methylphenidate.

### Quality Appraisal

Evaluation of each article against the CHEERS checklist criteria (Table S6) is provided in Table S7, Supplementary Material 4. Studies were consistent in reporting most checklist items, but no study reported all items. All studies adequately reported elements relating to title, background, target population, setting, estimating resources and costs, effects, discount rate, and assumptions. The most poorly reported items were related to a structured summary in the abstract, identification of model choice, heterogeneity, and currency, including date and conversion. Six studies did not give reasons for using a specific type of decision-analytical model.^38^,^39^,^41–43^,^46^ The study conducted by Maia, Stella, Wagner, Pianca, Krieger, Cruz, Polanczyk, Rohde and Polanczyk^35^ was the only one to report about characterising heterogeneity, by creating the model with two hypothetical cohorts of ADHD patients (children and adolescents), but no other confounding factors were assessed.

## Discussion

Despite heterogeneity in economic modelling methods used, 11 of the 12 included studies produced findings indicating that interventions intended to support neurodevelopment were cost-effective while improving quality of life for children and adolescents with neurodevelopmental delay or disability across four diagnostic groups. Within any economic modelling activity, there is a balance between appropriately simplifying complex health states and interventions to enable model parameterisation, without over-simplification to the point where findings are no longer representative of the underlying real-world context. Markov and decision tree models (or a combination of both) were used widely among included studies; however, no study was identified that applied time-to-event estimates in the context of discrete event simulation or microsimulation. The consistent choice of investigators to use cohort-based Markov health-state transition models or decision tree models rather than micro simulation highlights an ongoing challenge in the field of modelling costs and effects related to neurodevelopmental screening and intervention. Despite computational advances, the complexity associated with inter-dependencies in health-related conditions, sequalae, and multidisciplinary interventions can make assumptions regarding causality and nuanced time-to-events in long sequences difficult to parameterise in the absence of large robust longitudinal datasets from which this information can be drawn.^47^ Nonetheless, the authors of the identified studies are to be broadly commended for their application of economic methods in this challenging emergent field of economic evaluation and the relatively high standard of reporting observed.

The reporting quality of the included studies may be considered generally favourable when evaluated against the CHEERS criteria, with all studies reporting most of the recommended criteria (Table S7). Costing perspective was consistently reported across the included studies with a societal perspective most often adopted (10/12 studies), although more than half of studies evaluated pharmaceutical or clinical interventions, including medication management, surveillance management, and surgery. A societal perspective was likely appropriate in these studies since neurodevelopmental care is complex and often focuses on non-health metrics, including academic achievement,^18^ rate of employment,^19^ or social and physical capacities.^20^ The greatest opportunities for improving reporting quality relate to more transparent reporting of currency, price date and conversion, as well as characterising heterogeneity.

Measuring effectiveness of neurodevelopmental care is inherently challenging. It involves the assessment of physical, psychological, behavioural, social, and cognitive domains and the need to capture non-health benefits. Studies included in this review measured health benefits using QALYs (n=9), DFLYs (n=2), and LYs of severe delinquent behaviour prevented (n=1). Compared to direct approaches, these indirect approaches of utility measurement are arguably more appropriate to measure the health status of children with neurodevelopmental disorders for the purpose of economic modelling, as problems with language use and understanding questionnaires are commonplace within this population;^48^,^49^ however, deriving parameter estimates related to indirect measures of utility among children with neurodevelopmental delay or disability can also be challenging. The use of multi-attribute utility instruments to derive utility values of children’s health states in economic models in healthcare is more complicated than generating equivalent estimates in adults. There are constraints in terms of age-appropriateness, domains included, and methods used to derive utilities,^50^ among other challenges. The reported utility values in the reviewed studies were estimated using indirect approaches from multi-attribute utility instruments including the EQ-5D and HUI and primarily derived from published literature. Only two studies assessed and estimated utility values directly.^35^,^39^ A potentially important opportunity to advance derivation of utility values in the field is through the development and use of disease-specific or condition-specific multi-attribute utility instruments (MAUIs) appropriate for neurodevelopmental disorders.^10^ In addition, Sampaio, Feldman, Lavelle and Skokauskas^10^ have highlighted that existing MAUIs for young populations, including the Assessment of Quality of Life 6 Dimensions (AQoL-6D), the Child Health Utility 9 Dimensions (CHU9D), 16D, and 17D only cover a few aspects related to mental health, and no multi-attribute utility instrument is designed to measure health-related quality of life in children under age five years.^10^ Another study found that the Assessment of Quality of Life 8 Dimension Scale (AQoL-8D) to be more sensitive measure than others within context of economic evaluation of psychological interventions in melanoma.^51^ Despite numerous challenges associated with collecting quality of life information in young and medically complex populations in a way that is suitable for inclusion in economic modelling, there remains extensive opportunity for improvement in these estimates.

Communication deficits and cognitive disabilities can lead to difficulties measuring health outcomes in some children with neurodevelopmental disorders.^49^ Proxy reporting by caregivers has been widely used but may mask the lived experiences and perceptions of children themselves. Consequently, a combination of self-report and parent-proxy reported health utility values offer another potential solution for better-representing health utility values for young children with neurodevelopmental disorders. Only two of the 12 included studies used health utility values based on both self- and parent-proxy report.^37^,^40^ We recommend future research focus on developing and employing instruments to capture meaningful changes in outcomes for children with neurodevelopmental disorders, from their perspective. The current review allows us to identify health states and utility values (Table 3) that could be useful in future cost-effectiveness studies that evaluate pharmaceutical interventions for children with ADHD. However, this information was derived from five studies from a limited number of countries.

Non-health outcomes remain a challenge for inclusion in economic modelling in neurodevelopment. It is intuitive that non-health benefits are important and should be counted, including spill-over effects to other sectors (such as education, occupation, welfare, child protection, and justice systems) and to other people (including family members and educators). None of the reviewed studies assessed the impact of neurodevelopmental disorders on non-health benefits or caregivers’ health and quality of life, despite the documented consequences, especially in terms of caregiver mental health.^21^ An important recommendation arising from this review is that future studies at least consider the significant impact on caregivers’ quality of life and ideally include outcomes associated with this role in future economic evaluations. Health economists have started investigating how the inclusion of third-party burden effects on families in a cost-utility analysis may influence the reported value of adult mental health interventions^52^ and paediatric interventions mainly targeting infectious disease.^53^ Of particular relevance to neurodevelopment, a review of family effects in paediatric cost-utility analyses more broadly concluded that inclusion of third-party effects on family caregivers tend to make results more favourable.^53^ The potential use of discrete choice experiments offer an alternative solution for capturing health and non-health benefits to children, family members, and educators.^54^

For children with neurodevelopmental disorders, a lifespan time-horizon can be important because the impact of care is expected to manifest into adulthood. Consequently, this review excluded economic evaluation within a short analytical time horizon (less than 12 months). Except for Maia, Stella, Wagner, Pianca, Krieger, Cruz, Polanczyk, Rohde and Polanczyk,^35^ all other included studies have used 10 years or more as the time-horizon, with only one study applying a lifetime horizon for the decision analytic approach.^45^ In the absence of prospective studies that have followed up patient cohorts over long time-horizons, lifetime models are required to make substantial assumptions requiring the acknowledgement of considerable uncertainty in long-term model parameters. These model and parameter uncertainties contribute to overall uncertainty in models that have used longer time-horizons relative to models that have used shorter time-horizons. The potential impact of this uncertainty can be quantified through scenario and probabilistic sensitivity analyses which remain important considerations for future economic evaluations in the field. Similarly, modelling approaches that can appropriately account for recurring health states over longer time-horizons, including discrete event simulations and Markov models,^55^ are likely to be beneficial for future economic evaluations.

The emergent economic evaluations identified among a selection of common neurodevelopmental conditions (ADHD, ASD, cerebral palsy, dyslexia) are encouraging;^11–14^ however, the absence of economic evaluations in other diagnostic groups, as well as screening and intervention evaluation for neurodevelopmental delay or disability among important non-neurology-specific paediatric illness, including prematurity and congenital heart disease, remains a priority for research. For example, congenital heart disease has an incidence of 9.1 per 1000 live births^56^ and is associated with an increasing burden of neurodevelopmental delay or disability among these children who are now, due to medical and surgical advances, expected to live into adulthood. Economic evaluation of care models that integrate neurodevelopment evaluation, intervention, treatment, support, and follow-up care alongside cardiac care are likely to provide valuable information to improve clinical care and health policy for this at-risk patient population. This review has outlined the current methodological foundation and identified opportunities to extend the field of economic evaluations for neurodevelopment enhancing care models.

## Strengths and Limitations

A strength of this review included following the well-established Arksey and O’Malley framework^29^ for scoping reviews. This framework encourages researchers and clinicians to be engaged collaboratively in the review process.^29^,^30^ Consequently, stakeholders including clinicians from multiple disciplines were involved in identifying and developing the overarching research question, as well as key terms to identify relevant studies investigating common neurodevelopmental disorders. A core limitation of this review was that there were relatively few studies to review, with a strong focus on ADHD and a lack of studies including children with communication, motor, or tic disorders, or intellectual disability, thus restricting the generalizability of the findings while successfully highlighting priority areas for future research.

## Conclusion

This review has mapped health economic models used in the evaluation of neurodevelopmental care. While economic analyses in this field are currently scarce, emergent data from common neurodevelopmental disorders was encouraging in the quest for cost-effective care that improves quality of life among these conditions and was often found to be cost-saving. This review has provided a framework for future health sector modelling of neurodevelopmental care, which is a growing priority across many areas of paediatrics. Future work should not only expand on the work being done in common neurodevelopmental disorders but also examine neurodevelopmental care secondary to other health conditions, such as congenital heart disease.
